# Optimization of matrix assisted desorption/ionization time of flight mass spectrometry (MALDI-TOF-MS) for the characterization of *Bacillus* and *Brevibacillus* species

**DOI:** 10.1016/j.aca.2014.06.032

**Published:** 2014-08-20

**Authors:** Najla AlMasoud, Yun Xu, Nicoletta Nicolaou, Royston Goodacre

**Affiliations:** School of Chemistry and Manchester Institute of Biotechnology, University of Manchester, 131 Princess Street, Manchester, M1 7DN, UK

**Keywords:** Optimization, Bacteria, Mass spectrometry, Alignment, Classification

## Abstract

Over the past few decades there has been an increased interest in using various analytical techniques for detecting and identifying microorganisms. More recently there has been an explosion in the application of matrix assisted laser desorption ionization time of flight mass spectrometry (MALDI-TOF-MS) for bacterial characterization, and here we optimize this approach in order to generate reproducible MS data from bacteria belonging to the genera *Bacillus* and *Brevibacillus*. Unfortunately MALDI-TOF-MS generates large amounts of data and is prone to instrumental drift. To overcome these challenges we have developed a preprocessing pipeline that includes baseline correction, peak alignment followed by peak picking that in combination significantly reduces the dimensionality of the MS spectra and corrects for instrument drift. Following this two different prediction models were used which are based on support vector machines and these generated satisfactory prediction accuracies of approximately 90%.

## Highlights

• Optimization of MALDI-TOF-MS for characterizing *Bacillus* and *Brevibacillus* species.
• Development of a suitable chemometric workflow for processing raw MALDI-TOF-MS data.
• Classification of 7 species from bacteria achieved high accuracy (∼90%).
• Allowed to dry at room temperature (*ca*. 22 °C) for 1 h.

## Graphical abstract

**Figure:**
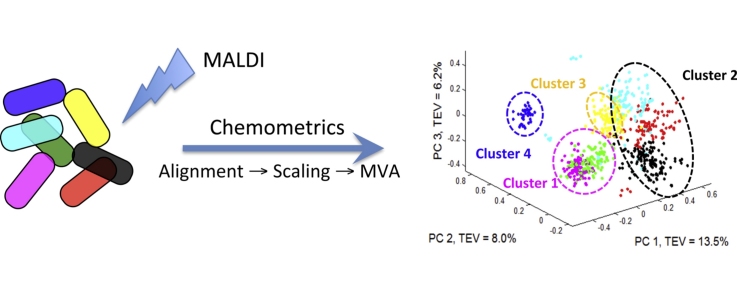

## 1 Introduction

*Bacillus* are rod-shaped aerobic Gram-positive bacteria that are able to sporulate. These bacteria are normally found in the soil, plants, and can be transferred to meat and dairy products where they can spoil food making them unfit for human consumption [1]. Even though most of these bacteria are harmless saprophytes there still remains a few toxic members of this genus, such as *Bacillus subtilis* and *Bacillus cereus*, which are often associated with food-borne infections [2], along with the more notorious *Bacillus anthracis* the casual agent of anthrax. Whilst *Bacillus sphaericus* is toxic to insects and is used for biocontrol of mosquitoes [3]. *B. subtilis* is the most scientifically defined member of the *Bacillus* genus and has thus been used as a model organism in many genetic research studies. Other members of this *B. subtilis* group are less defined and are harder to identify such as *Bacillus licheniformis* and *Bacillus amyloliquefaciens* because they are very similar microorganisms [1], [4]. The *B. cereus* group contains a number of different bacteria, with some leading to negative health implications in humans, and as discussed above have sometimes been linked to food poisoning [5], [6], [7].

The unequivocal identification of bacterial is a vital step in medical therapy and the food industry and this is usually performed at the genotypic or phenotypic level. A number of traditional methods have so far been used to identify microorganisms, such as cell culturing with differential staining [8], polymerase chain reaction (PCR) [9], [10], [11], [12] and enzyme linked immunosorbent assays (ELISA) [13]. Whilst these approaches formed the foundations of knowledge and understanding in microorganism research, these methods are very time consuming, costly and labour intensive, hence more rapid detection methods are continually needed [14]. In addition to rapid testing, methods that provide molecular-specific information are also preferred as these may allow one to relate any markers to specific microbiological function.

Modern methods for the identification of microorganisms have recently focussed on mass spectrometry as these are rapid and provide molecular information on the bacteria under investigation. Whilst pyrolysis mass spectrometry was used for bacterial analysis in the past [15], current methods are based on electrospray-ionization (ESI-MS) [16], [17] and the more popular method of matrix-assisted laser desorption ionization (MALDI-MS) [14], [18], [19], [20]. MALDI-TOF-MS is easy to use, provides rapid results, and has been used for identification and taxonomy of microorganisms [18], [21], [22]. The maturity of this analytical technique has benefitted its application to a wide range of areas such as proteomics [23], [24], [25], intact-cell mass spectrometry (ICMS) [19], [26], [27], [28], [29] and in the area of lipidomics [30], [31], [32].

MALDI-MS on bacteria (and indeed other complex samples) results in a multivariate spectral pattern, which usually provides information on the protein content of the bacterium under analysis. This protein profile or barcode can be matched against MALDI-MS profiles/barcodes that have been previously collected under identical conditions and stored within (usually) organism specific databases [22], [23], [33], [34]. This matching may involve the generation of dendrograms from hierarchical cluster analyses (HCA) [33], [35] or ordination plots from principal component analysis (PCA) [36], [37] or discriminant analysis (DA) [38], [39].

The aim of this study was to generate a reproducible MALDI-TOF-MS protocol for measuring the protein spectra from bacteria. In order to establish this we used a set of 34 well-characterised bacteria belonging to the genus *Bacillus*. In a series of experiments we optimised the matrix and the sample preparation method used using first a mixture of pure proteins followed by the analysis of a subset of these bacilli, before the optimised method was used on the full set of 34 bacteria.

## 2 Materials and methods

### 2.1 Compounds

Trifluoroacetic acid (TFA), acetonitrile (ACN), sinapinic acid (SA), caffeic acid (CA), 2,5-dihydroxybenzoic acid (DHB), α-cyano-4-hydroxycinnamic acid (CHAH), ferulic acid (FA), 2,4,6-trihydroxyacetophenone monohydrate (THAP), 2-(4-hydroxyphenylazo)benzoic acid (HABA), 2,6-dihydroxyacatophenone (DHAP), 9-aminoacridine (9-AA) and dithranol (INN) from Sigma–Aldrich (Dorset, UK) were used.

14 g of nutrient agar (Fisher Scientific Ltd. Loughborough, UK) was dissolved and mixed thoroughly in a bottle containing 500 mL of water. This bottle was then autoclaved at 121 °C for 15 min and subsequently used for the bacterial cultures.

### 2.2 Standard protein samples for MALDI-TOF-MS

Five different proteins were mixed together at the same concentration (20 μM) to find the optimum matrix and deposition method for pure protein analysis. These proteins (molecular weight provided in parentheses) included: insulin (5735), cytochrome *c* (12,362), apomyoglobin (16,952), aldolase (39,212) and albumin (66,430) and were acquired from Sigma–Aldrich.

### 2.3 Bacterial culturing

General information of the 34 strains of *Bacillus* is provided in Table 1 and these belonged to two genera (*Bacillus* and *Brevibacillus*) and seven different species. The cells were cultured on nutrient agar and were incubated at 37 °C for 24 h. Bacterial strains were cultivated aerobically three times under these conditions to make sure that the cultures were axenic, and to maintain a stable phenotype. After this was established single bacterial colonies were then cultured on nutrient agar and also incubated at 37 °C for 24 h. Five biological replicates were prepared for each isolate. After growth the biomass of each sample was carefully collected using two full sterilised plastic loops (equivalent to about 20 μL). This biomass was then centrifuged for 3 min at 13,000 ×* g*. The pellets containing the bacteria were then washed twice with 1 mL of distilled water to remove residual culture media, centrifuged again to remove the supernatant, and the pellet was then stored at −80 °C until further analysis.

**Table 1:** The 34 *Bacillus* species and strains used in this work.

Sample no.	Species	Strain no.	Key colour used in figures
1	*B. sphaericus*	7134^T^	Yellow
2		B0408*	
3		B0219	
4		B0769	
5		B1147	

6	*Br. laterosporus*	B0043	Blue
7		B0262	

8	*B. subtilis*	B0014^T^, *	Black
9		B0044	
10		B0098	
11		B0099	
12		B0410	
13		B0501	
14		B1382	

15	*B. cereus*	B0002^T^, *	Green
16		B0550	
17		B0702	
18		B0712	
19		B0851	

20	*B. amyloliquefaciens*	B0177^T^	Red
21		B0168*	
22		B0175	
23		B0251	
24		B0620	

25	*B. megaterium*	B0010^T^, *	Pink
26		B0056	
27		B0057	
28		B0076	
29		B0621	

30	*B. licheniformis*	B0252^T^, *	Cyan
31		B0242	
32		B0755	
33		B1081	
34		B1379	

T Indicates the type strain.

* Indicates strains used for preliminary optimization experiments.

### 2.4 Optimization of MALDI-TOF-MS

Optimization of sample preparation was carried out in order to identify the most appropriate matrix preparation and deposition method for the analysis of bacteria. Initial experiments optimised the matrix and deposition method on mixtures of pure proteins (Supplementary Information Table S1 illustrates the four different sample preparation methods for MALDI-TOF-MS). Briefly, 10 different matrices were used to find the most compatible matrix for MALDI-TOF-MS analysis and these included DHB, CHAH, SA, FA, THAP, CA, HABA, DHAP, 9-AA and INN. At the same time four different depositions methods (mix, overlay, underlay and sandwich) were investigated for protein sample preparation. The optimised conditions involved using SA as the matrix and the mix method for sample deposition and this was subsequently used for bacterial analysis. We note of course that the five proteins chosen are a substitute for bacterial analysis and we did not assume that the best protein preparation method would be the optimal method for bacteria so we tested the top three matrices and preparation methods on a small subset of bacteria (the type of strain from each species is marked with ‘T’ and the strains used for preliminary optimization experiments were marked with ‘*’ in Table 1); SA with the mix method was indeed the best method (data not shown for this optimization).

### 2.5 Bacterial sample preparation

Preliminary experiments also suggested that it was important to optimise the appropriate amount of biomass for MALDI-MS; which one can think of as the amount of matrix:analyte ratio. The defrosted pellet from above (which contained ∼10^10^ CFU (colony forming units)) was diluted at various levels in water containing 0.1% TFA (250, 500, 1000, 1500 and 4000 μL; data not shown except for 1000 μL water containing 0.1% TFA). The optimum pellet dilution was established at 1000 μL and this was subsequently used.

For MALDI-TOF-MS analysis of the bacteria 10 mg SA was dissolved in 500 μL of ACN and 500 μL of water containing 2% TFA. 10 μL from the above bacterial sample and 10 μL of matrix were mixed together (Table S1) and vortexed for 10 s before. 2 μL from the resultant mixture was spotted on a MALDI-TOF-MS stainless steel target plate. This was allowed to dry at room temperature (*ca*. 22 °C) for 1 h.

### 2.6 MALDI-TOF-MS

Samples were analysed in batches using an AXIMA-Confidence (Shimadzu Biotech, Manchester, UK) mass spectrometer. This MALDI-TOF-MS device contained a nitrogen pulsed UV laser with a wavelength of 337 nm as described previously [40]. The power of the laser at the laser head used was set to 140 mV. Each profile contained 20 shots, and 100 profiles were collected using a circular raster pattern. The MS was operated in positive ion source and linear TOF was used over the range from 1000 to 80,000 *m*/*z*. The collection time for each sample was ∼3 min and each biological sample was analysed four times (technical replicates). A single biological replicate for each of the 34 bacteria was analysed each day, and the analysis time took 5 days of machine time during a 2 week period. The result of this analysis generated 680 MALDI-TOF-MS spectra: 34 bacteria × 5 biological replicates × 4 technical replicates. The MALDI device was calibrated using the protein mixture mentioned above.

## 3 Data analysis

### 3.1 Pre-processing

MATLAB 2010a (The Math Works, Natick, MA, USA) was used for pre-processing and data analysis. Baseline corrections were first performed on the spectra by using asymmetric least squares (AsLS) [41]. In addition, the interpolation and alignment of MALDI-TOF-MS spectra in the *m*/*z* axis were required in order to integrate all the spectra in a unified coordinate system and also reduce the amount of ambiguities of assigning peaks from different samples collected over the 2 week period (see below). This was achieved by firstly interpolating all the spectra into a common *m*/*z* domain which is from 1000 to 13,000 *m*/*z* with an interval of 0.1078 *m*/*z* and then an algorithm named interval correlation optimized shifting (icoshift) [42] was used to correct *m*/*z* drifting across different samples. Peak picking was then performed on the aligned spectra to detect mass peaks in each spectrum and this was performed using intensity weighted variance (IWV) algorithm as described by Jarman et al. [43]. The detected peaks of all the samples were then aligned together with a drift tolerance threshold of ±1 *m*/*z*. After this peak picking and alignment process, a total number of 243 unique mass peaks were detected and resulted in a peak table matrix of dimensions 680 × 243 which was used for further data analysis. The peak intensities were firstly log_10_-scaled and then normalised so that the sum of squares of each row (i.e. a sample) equals 1.

### 3.2 Multivariate analysis

Two different types of analysis were performed on the data: one was a semi-quantitative analysis and the other a qualitative analysis.

The semi-quantitative analysis was performed on the log_10_-scaled and normalised peak intensity table matrix. Principal components analysis (PCA) was performed first to reveal the ‘natural’ pattern of the data and then support vector machines (SVM), with a linear kernel, was used for supervised classification. The SVM models were validated by using a bootstrap replacement procedure coupled with cross-validation for the model parameter selection (see below). In this process the data were first split into a training set and a test set via a bootstrapping resampling based on the biological replicates; i.e. all the samples from the same biological replicates were considered as one during the resampling. Considering the random nature of this bootstrapping process, the number of samples selected in the training and test sets varied between the different 1000 iterations, on average 63.3% of the samples would be in the training set and 36.7% in the test. Next a *k*-fold cross-validation was performed on the training set where *k* is the number of unique biological replicates in the training set, the error penalty parameter *C* within the SVM varied from 1 to 10^6^ and the one which yielded the lowest cross-validation error was chosen to build the SVM model. The model was then applied to the test set generated via the bootstrapping selection in order to calculate the predictive accuracy of the test set. This bootstrap procedure was repeated 1000 times and the collected predictive accuracies for the test set only were then averaged. This can be considered as an unbiased estimation of the generalisation performance of the SVM model. Two types of classification were carried out: one was to classify the samples on species level (7 classes); and the other was to classify the samples on strain level (34 classes). Both types of classification followed the same validation procedure as described above.

The qualitative analysis on the data focused on the presence/absence of certain feature (i.e. mass peaks) while ignoring the intensities of the peaks. The peak table matrix was converted into a binary format: if a peak had been detected in one particular sample the corresponding element in the matrix was set to 1 and 0 if otherwise; the threshold for presence/absence was set to be 3× standard deviation of baseline signals. Principal coordinate analysis (PCoA) was used as a counterpart of PCA in the qualitative analysis and the Jaccard distance was used to measure the dissimilarity between the samples. A distance matrix ***D*** was calculated which contains the Jaccard distance between every pair of samples. PCoA was then applied to ***D*** to obtain a scores matrix and this scores matrix can be interpreted in the same way as the scores matrix obtained from PCA. For supervised classification, a naïve Bayesian classifier and SVM with a Jaccard kernel [44] were applied to the data. Both classifiers were validated using exactly the same bootstrapping procedure as described above and the classifications were again performed on both species and strain level.

## 4 Results and discussion

### 4.1 MALDI-TOF-MS optimization

Initially a mixture contain five different proteins was used to obtain the optimum conditions for protein analysis using MALDI-TOF-MS. At this stage 10 matrices were used to determine the most suitable matrix and four sample preparation procedure when performed. Good protein detection was seen for SA, CA and FA, whilst others such as DHAP and 9-AA were not suitable matrices for protein analysis. Results obtained from this study showed that SA was the most suitable matrix for protein analysis (Tables S2–S5). This finding was supported by other workers analysis [36], [45], [46], [47], [48], and this may be due its classification as a hot matrix, which causes less protein fragmentation [49]. In addition, as discussed by Vaidyanathan [24], the reason behind SA’s compatibility lies in its high level of homogeneity and crystallisation with the solvent when SA is mixed with bacteria.

During the matrix optimization the most appropriate sample deposition method for protein analysis was also assessed. Four methods were used (see Table S1 for details) and it was found that the ‘mix method’ where sample and matrix are pre-mixed prior to spotting on the MALDI target plate was best. This deposition method was very reproducible and caused improved desorption and ionization in comparison with other deposition methods. Tables S2–S5 (see SI) summarises the data obtained from analysing the 5-way protein mixture using the 10 different matrices and the 4 different deposition methods.

After this the top 3 matrices (SA, CA and FA) were assessed on a subset of 6 bacteria comprising the type strain from each species. SA with the mix method was also the best method in terms of the number of protein peaks routinely detected in replicate analyses and in terms of the reproducibility of signal (as judged by PCA; data not shown). Thus SA with the mix method was used for all bacterial analyses.

### 4.2 Bacillus MALDI-TOF-MS spectra

**Fig. 1:**
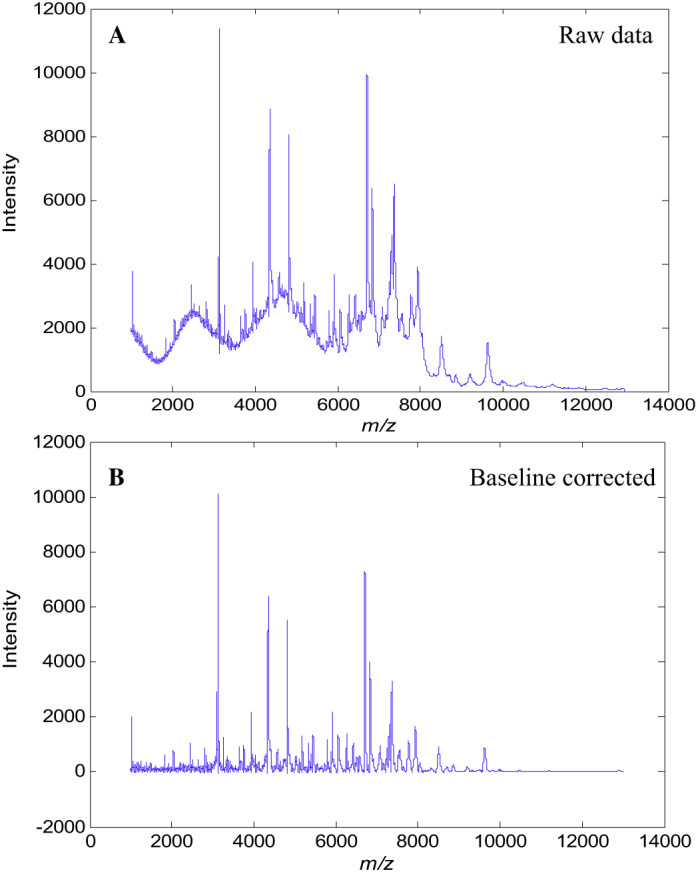
Differences between MALDI mass spectra obtained from the analysis of *B. cereus* B0712 (A) before and (B) after baseline correction.

**Fig. 2:**
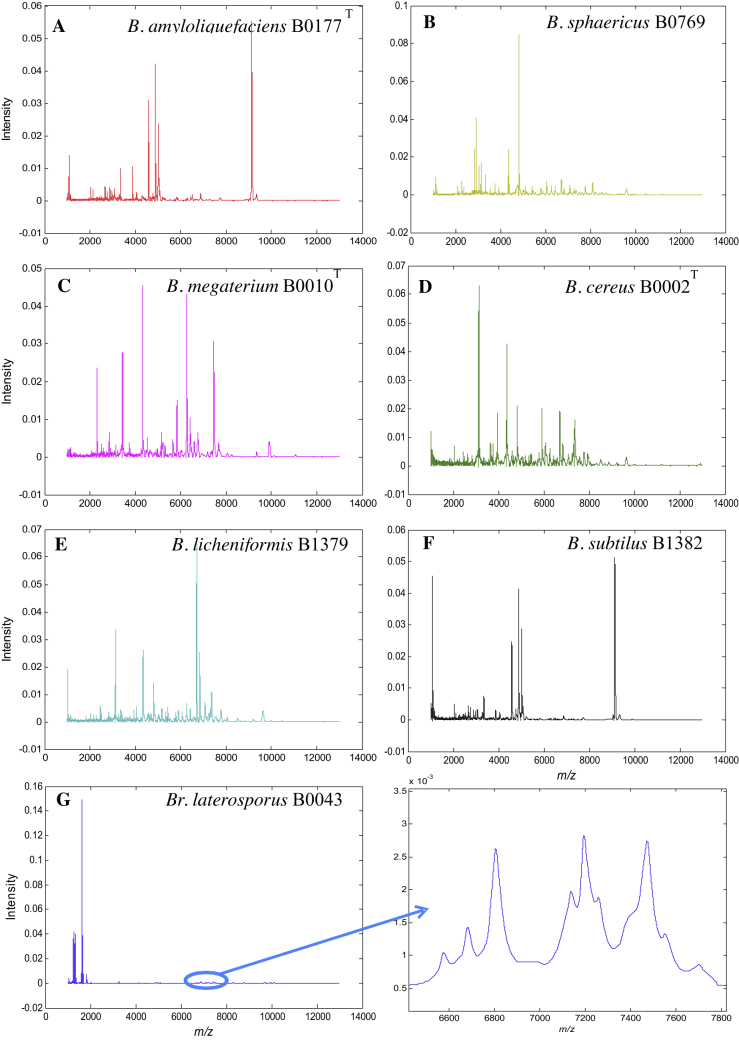
Typical MALDI-TOF-MS spectra of (A) *B. amyloliquefaciens* B0177, (B) *B. sphaericus* B0769, (C) *B. megaterium* B0010^T^, (D) *B. cereus* B0002, (E) *B. licheniformis* B1379, (F) *B. subtilus* B1382 and (G) *Br. laterosporus* B0034. The panel to the right of (G) is a zoomed in region (highlighted with an ellipse) of the MALDI-TOF-MS spectrum from *Br. laterosporus* B0034. These spectra have been baseline corrected and normalized, so that the sum of each squared spectrum equals to 1.

Typical MALDI-TOF-MS spectra of *B. cereus* B0712 obtained SA with the mix method for both the raw MS data and after baseline correction and alignment are shown in Fig. 1. It is clear from the raw data from this bacterium (and indeed all the bacteria analysed; data not shown) that significant baseline artefacts are observed which were unavoidable. Spectra were therefore pre-processed using the following routine: (i) baseline correction was performed using AsLS on the raw MS profiles; (ii) this was followed by spectral alignment using icoshift (Fig. 1) and (iii) finally, following this step these spectra were scaled so that the sum of square of each spectrum equals to 1. Typical normalised and scaled spectra of all 7 type strains from these bacilli are shown in Fig. 2A–G.

It is known that sample preparation for bacterial analysis is important and this has been discussed before for the analysis of *Bacillus* species [17], [29], [33]. It can be seen that these MALDI-TOF-MS spectra are generally distinct from one another and possess good signal-to-noise in the *m*/*z* 1000–13,000 range used. Whilst some spectra are clearly very different, *Brevibacillus laterosporus* (which belongs to a different genera) compared with the other *Bacillus* species, it is very difficult to use only visual inspection to identify these different bacteria. Therefore chemometric methods are needed for spectral analysis.

The spectra that were generated from MALDI-TOF-MS are very high dimensional nature and each spectrum contains 0.1078 *m*/*z* intervals after interpolation with ion counts at each value. It is clear from the spectra (Fig. 2) that much of this information is redundant (i.e. noise), such that direct computation using PCA would be both puerile, as many spurious correlations may be found, as well as being computational intense.

Therefore we used peak picking to select only those *m*/*z* which had arisen from real signals. In this process the intensity weighted variance (IWV) algorithm was used and resulted in a peak table comprising 243 features from the bacteria analysis of 680 samples. This matrix was of dimensions 680 × 243 and significantly reduced from the full spectra (680 × 111,339) and was used for further data analysis.

**Fig. 3:**
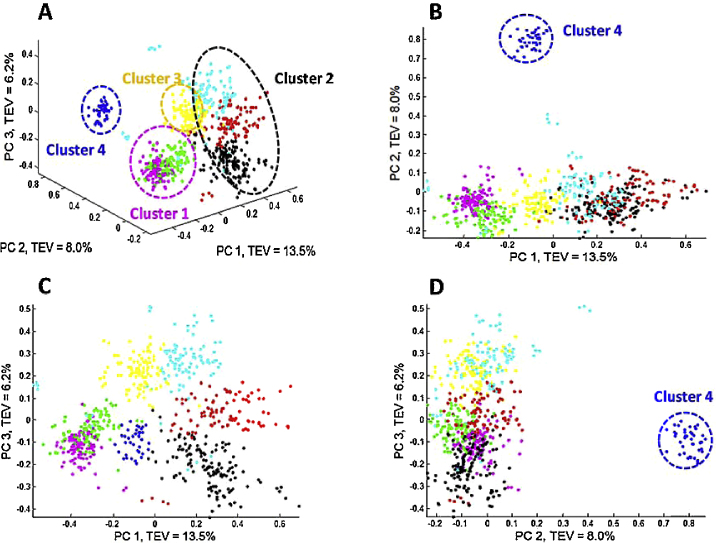
PCA scores plots from the peak table matrix after pre-processing the MS data. Multiple principal components are plotted: (A) PC1 *vs*. PC2 *vs*. PC3; (B) PC1 *vs*. PC2; (C) PC1 *vs*. PC3 and (D) PC2 *vs*. PC3. The colours represent the different species see Table 1 for annotations. TEV: total explained variance for the PC score plotted. (For interpretation of the references to colour in this figure legend, the reader is referred to the web version of this article.)

**Fig. 4:**
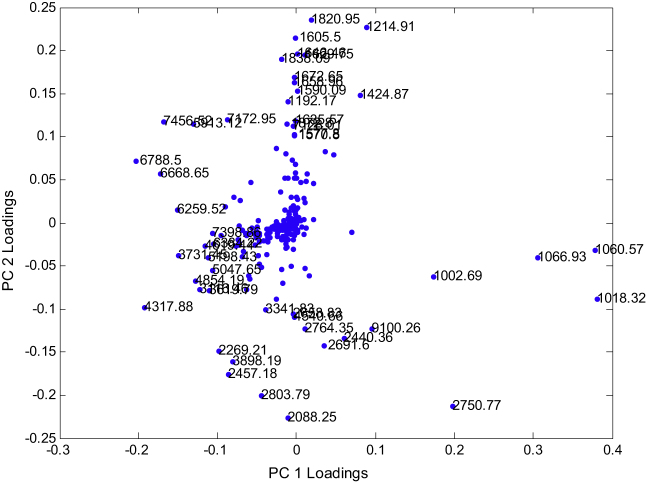
PCA Loadings plots from the peak table matrix after pre-processing the MS data.

The scores plots of the first 3 PCs from PCA performed on the peak table matrix are provided in Fig. 3 and the loadings plot of the first 2 PCs are provided in Fig. 4. The variables with their absolute loadings (either PC1 or PC2) greater than 0.1 are labelled in Fig. 4 along with their corresponding *m*/*z*. Four main clusters can be observed: (1) the first contained *Bacillus megaterium* and *B. cereus*; (2) comprised *B. subtilus*, *B. amyloliquefaciens* and *B. licheniformis*; (3) contained only *B. sphaericus*; and (4) was also a single member cluster of *Br. laterosporus* (see Fig. 3A for an annotated 3-D representation). The MALDI-TOF-MS spectra obtained from the analysis of *Br. laterosporus* (Fig. 2G) were very different to the spectra from the other *Bacillus* species and this was reflected in PCA clusters (Fig. 3). As can be seen *Br. laterosporus* strains were significantly different in PC2 (Fig. 3B and D) which is why when PC2 *vs.* PC3 were plotted the groupings of the other 3 clusters were revealed. This was perhaps not surprising as this species belonged to a different bacilli genus, namely *Brevibacillus*.

The reason for choosing this set of bacilli is that these species have previously been analysed using a range of classification approaches including miniaturised biochemical test Analytical Profile Index (API), genotyping using 16S rDNA sequencing and an alternative physciochemical methods to MALDI-MS called Raman spectroscopy that measures molecular vibrations of functional groups. Based on the API tests these bacteria have been placed into four different groups [50] consisting of: (I) *B. cereus*; (II) *Br. laterosporus*; (III) *B. sphaericus*; (IV) *B. megaterium*, *B. subtilis*, *B. licheniformis* and *B. amyloliquefaciens*. Slightly different clusters were also previously found from 16S rDNA analysis: clusters (I); (II) and (III) from the API were also seen, but the *B. subtilis* group (comprising *B. subtilis*, *B. licheniformis* and *B. amyloliquefaciens*) was split from *B. megaterium*; in addition, although clustered separated *B. cereus* and *B. megaterium* were relatively close relatives at the genetic level [28], [39]. The grouping generated from our MALDI-TOF-MS analysis is therefore highly congruent with both phenotypic (API) and phylogenetic markers (16S rDNA), as well as other biophysical characterization methods based on UV resonance Raman spectroscopy [39].

**Fig. 5:**
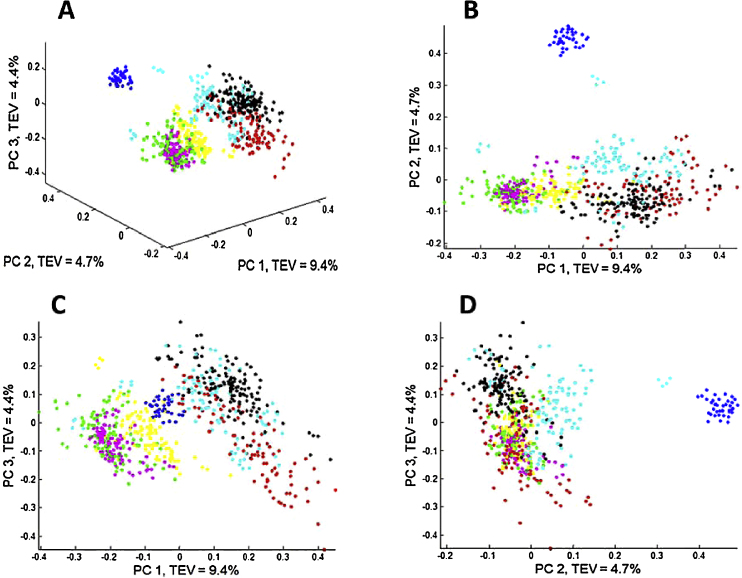
PCoA scores plots of the data obtained to show clusters of present and absent peaks using the Jaccard distance model. Multiple principal components are plotted: (A) PC1 *vs*. PC2 *vs*. PC3; (B) PC1 *vs*. PC2; (C) PC1 *vs*. PC3 and (D) PC2 *vs*. PC3. The colours represent the different species see Table 1 for annotations. TEV: total explained variance for the PC score plotted. (For interpretation of the references to colour in this figure legend, the reader is referred to the web version of this article.)

The results above used the quantitative data from the peak intensities, or at least the log_10_ of the signal to try and make the data appear normally distributed. In preliminary analyses we also attempted square root scaling and this produced similar results; for brevity we report only log_10_ here. As detailed in the materials and methods we also processed the data so that they were considered qualitative in nature; that is to say, we encoded the mass ions as being present (1) or absent (0). The purpose of employing such a strategy is to test whether such greatly simplified information is still sufficient to discriminate different types of bacteria, either on species level or strain level. Moreover, this would compensate for the fact that MALDI-TOF-MS is not considered truly quantitative. We, and others, have observed differences in the ion intensities of proteins from intact bacteria [19], [26] and this significant variation in the peak intensities can be due to various analytical reasons. These are most likely due to small changes in bacteria growth, sample handling and the formation of different co-crystals with the matrix ‘spot’ [51], [52]. If this qualitative approach were successful, it would suggest that the characterization of the bacteria based on the MALDI-TOF-MS spectra is in fact not sensitive to such variations and would suggest that MALDI-TOF-MS, as an analytical platform, is robust for bacterial analyses. Moreover, those features which had high probabilities of occurrence in some types of bacteria while absent or much rarer in other types could have significant biological implications and perhaps worth further investigation. Therefore PCoA was performed on the binary peak table matrix and resulted in a highly similar pattern (Fig. 5) to the one showed in the PCA scores plot (Fig. 3). This had suggested that based on the information of presence/absence of the features, it was indeed possible to discriminate bacteria on species level.

### 4.3 Automated identification of Bacillus from their MALDI-TOF-MS spectra

The next stage was to assess whether the information from the MALDI-TOF-MS data were discriminative enough to allow identification using supervised learning methods. The results of these classifications performed at the species level (i.e. 7 classes to be predicted) are given in Table 2, Table 3 using support vector machines (SVM) for the semi-quantitative and qualitative data, respectively. While prediction accuracies at the strain level (i.e. 34 class prediction) are provided in SI Tables S6 and S7.

**Table 2:** Prediction accuracies of the seven species from *Bacillus* using DAG-SVM with the linear kernel model.

	*B. am* (%)	*B. ce* (%)	*Br. la* (%)	*B. li* (%)	*B. me* (%)	*B. sp* (%)	*B. su* (%)
*B. am*	92.56	0.13	0.00	0.11	0.58	0.95	5.68

*B. ce*	3.37	83.37	0.00	0.12	11.27	1.82	0.05
*Br. la*	0.00	0.00	100.00	0.00	0.00	0.00	0.00
*B. li*	5.28	1.41	0.00	80.26	2.65	3.93	6.47
*B. me*	0.10	9.22	0.00	0.00	90.67	0.01	0.00
*B. sp*	1.37	1.91	0.00	2.41	0.09	94.23	0.01
*B. su*	6.46	0.00	0.00	2.13	0.00	0.00	91.42

*B*. *am*: *B. amyloliquefaciens*, *B. ce*: *B. cereus*, *Br. la*: *Br. laterosporus*, *B. li*: *B. licheniformis*, *B*. *me*: *B. megaterium*, *B. sp*: *B. sphaericus* and *B. su*: *B. subtilis*.

**Table 3:** Prediction accuracies of the seven species from *Bacillus* using DAG-SVM with the Jaccard kernel model.

	*B. am* (%)	*B. ce* (%)	*Br. la* (%)	*B. li* (%)	*B. me* (%)	*B. sp* (%)	*B. su* (%)
*B. am*	91.29	0.23	0.00	0.14	0.85	1.36	6.14

*B. ce*	3.09	81.75	0.00	0.02	12.26	2.67	0.22
*Br. la*	0.00	0.00	100.00	0.00	0.00	0.00	0.00
*B. li*	5.67	2.57	0.00	78.64	1.62	3.71	7.79
*B. me*	0.04	8.79	0.00	0.01	91.12	0.04	0.00
*B. sp*	1.06	4.17	0.00	1.98	0.30	92.45	0.05
*B. su*	4.23	0.00	0.00	1.46	0.00	0.00	94.31

*B*. *am*: *B. amyloliquefaciens*, *B. ce*: *B. cereus*, *Br. la*: *Br. laterosporus*, *B. li*: *B. licheniformis*, *B*. *me*: *B. megaterium*, *B. sp*: *B. sphaericus* and *B. su*: *B. subtilis*.

It is very interesting to see that the SVM with Jaccard kernel (i.e. the SVM model based on the presence/absence information) and the SVM with Linear kernel gave almost identical prediction accuracies. This suggests that the qualitative information on protein content is sufficient to effect accurate classification, rather than the level of the proteins in the bacterial cells.

For the species classification models, the SVM with a linear kernel had an average correct classification rate (CCR) of 89.27% and the SVM with the Jaccard kernel providing 88.92% average CCR. The naive Bayesian classifier accuracy was slightly worse (77.69% average CCR). For all classification models *Br. laterosporus* was never mis-classified which is perhaps unsurprising as it is a difference genus. *B. cereus* and *B. megaterium* were sometimes misclassified as each other, which was also to be expected as these are phylogenetically similar [50]. Finally, the *B. subtilis* group comprising *B. amyloliquefaciens*, *B. licheniformis* and *B. subtilis* which are similar at the biochemical and genetic level [53] were also occasionally misclassified as each other. If these were taken as a single group the classification for these three species (e.g. in Table 2) would increase from 91.29%, 78.64%, 94.31% to 97.57%, 92.10%, 100% for *B. amyloliquefaciens,* *B. licheniformis* and *B. subtilis*, respectively. The fact that such observations were consistent across all the classification models indicates this is a model independent general trend and a reflection of the phenotypic characteristics being measured using MALDI-TOF-MS.

The CCRs of the classification models for strain (*n *= 34) classification is as expected much worse than those at the species level. The average CCR for these models ranged from 45.88% to 54.04% (SI Tables S7 and S6) for the qualitative and semi-quantitative models. As expected the misclassification of these bacterial strains usually occurred within the same species but to different strains. These may seem poor but considering the fact that there were 34 strains analysed this is a large number of classes and the expected CCR from a random classification model would be only 2.9%. Therefore the prediction accuracies of these models were still very impressive. It was also notable that the semi-quantitative classifier was ∼9% better than the qualitative model which suggests that unlike the species classification the information on the peak intensities might also be required to achieve better discrimination between the strains.

## 5 Concluding remarks

MALDI-TOF-MS is gaining popularity for microbial classification and identification [54], [55], [56], [57]. This results in information on the protein content of the organism under study and this proteomic barcode can be used to characterise the bacteria under investigation. However, in order to generate a consistent barcode the analytical approach must be optimised and tested. In this study we assessed 10 different matrices with 4 different sample preparation approaches. These 40 conditions were first applied to protein mixtures and the top 3 matrices-preparation methods were then assessed for reproducibility and for the generation of information rich protein profiles on 6 bacteria. This established that sinapinic acid with the mixed sample preparation approach was the preferred method, which is in agreement with other studies [46], [58].

This matrix was then used on all 34 bacilli and each bacteria was grown 5 times and each of these biological replicates were analysed 4 times (technical replicates). These 680 MALDI-TOF-MS spectra were collected over a period of 2 weeks. Due to the extended mass range over which the spectra were collected (1000–13,000 *m*/*z*) significant drift in the *m*/*z* X-axis was observed which if not corrected would adversely affect bacterial characterization. This was successfully overcome by aligning the peaks using interval correlation optimized shifting. Preprocessing also involved using asymmetric least squares for baseline removal. Chemometric classifiers were then used on these data and the same data after peak picking using intensity weighted variance. This peak picking reduced the dimensionality of the MS data from a massive 680 samples × 111,339 *m*/*z* channels (75,710,520 data points) to a mere 680 × 243 (165,240 data points) and this process did not negatively affect classification.

Classification accuracies at *Bacillus* species level were ∼90% for the 7 species under analysis and this was robustly tested using bootstrap analysis. The few misclassifications that were made could be readily explained by very close species similarity of the *B. subtilis* group (*viz*. *B. amyloliquefaciens*, *B. licheniformis* and *B. subtilis*). In conclusion we have developed a robust MALDI-TOF-MS data collection and data analysis pipeline that we shall now expand to the analysis of other bacterial groups.
