# Supplementary material for: Optimization of matrix assisted desorption/ionization time of flight mass spectrometry (MALDI-TOF-MS) for the characterization of Bacillus and Brevibacillus species
Source: Anal Chim Acta. 2014 Aug 20;840:49–57. doi: 10.1016/j.aca.2014.06.032 (PMC4223412; doi:10.1016/j.aca.2014.06.032)
Supplement: Supplementary file 1 [file mmc1.docx]

**SUPPLEMENTARY INFORMATION**

**Optimization of matrix assisted desorption/ionization time of flight mass spectrometry (MALDI-TOF-MS) for the characterization of *Bacillus* and *Brevibacillus* species**

**Najla AlMasoud, Yun Xu, Nicoletta Nicolaou and Royston Goodacre**

School of Chemistry, Manchester Institute of Biotechnology, The University of Manchester, 131 Princess Street , Manchester, M1 7DN, UK.

Correspondence to Prof Roy Goodacre: [roy.goodacre@manchester.ac.uk](mailto:roy.goodacre@manchester.ac.uk)

**Tel:**+44 **(**0) 161 306-4480

**Data Processing and Peak Picking**

As these data were collected over a relatively long period we also needed to develop spectra pre-processing and peak picking algorithms that allowed robust and reproducible profiles to be generated. This process is detailed below:

In liquid chromatography–mass spectrometry (LC-MS) or gas chromatography–mass spectrometry (GC-MS) any chromatographic shifts (due to unavoidable changes in retention times of analytes that are being separated) can be aligned based on the mass spectrum and the results of such alignment checked (indeed guided) using the unique fragmentation of the analytes within the aligned spectra. However such orthogonal data do not exist in MALDI-TOF-MS as generally no fragmentation is used; indeed even with TOF-TOF configurations this would not be possible due to the large *m*/*z* used. Thus it is not possible analytically to establish if our alignment and peak picking process was successful, and excessive misalignment of the peaks could have undesired effect on the ability to effect accurate bacterial identification from the MALDI-TOF-MS spectra. We therefore performed PCA on both the log_10_-scaled peak table matrix and the log_10_-scaled raw spectra. The results showed that the scores plot obtained from the PCA performed on the peak table matrix were highly similar to that obtained from the raw data (data not shown). To quantify the level of similarity Procrustes analysis [1] was performed on the two sets of PC scores using the first 3 PCs and a Procrustes error of 0.2474 was obtained. Given there were 680 samples and merely 3 variables (PC scores), such Procrustes error is considered very low. For comparison, if the order of the samples was randomly permuted the Procrustes error was always greater than 0.99. Thus we can conclude that the patterns represented by these two types of data are highly comparable and this suggests that the information in the raw data had indeed been faithfully translated to the very much smaller peak table matrix.

[1] J.C. Gower, G.B. Dijksterhuis, Procrustes problems, Oxford University Press Oxford, 2004.

**Table S1** Details of the four different sample preparation methods for MALDI-TOF-MS (A) Mix, (B) overlay, (C) underlay and (D) sandwich.

| **Deposition method** | **Sample preparation** |
| --- | --- |
| **(A) Mix**  **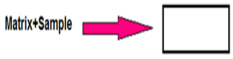** | 10 µL of the prepared protein mixture were added to 10 µL of each matrix in an Eppendorf tube. The sample was then mixed by vortexing to ensure thorough mixing. 2 µL of the resultant matrix/protein mixture was applied to the MALDI plate and allowed to dry. Once the liquid had evaporated the plate was then ready for analysis. |
| **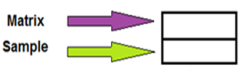(B) Overlay** | 1 µL of the protein mix sample was applied to the MALDI plate and was allowed to dry. Following evaporation, 1 µL of matrix was added to the protein sample. |
| **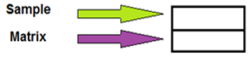(C) Underlay** | 1 µL of matrix was applied to the MALDI plate and was allowed to dry. Following evaporation, 1 µL of the protein mix sample was added to the matrix. |
| **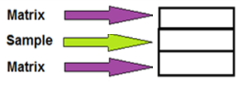(D) Sandwich** | 0.5 µL of matrix was applied to the MALDI plate and was then removed. 1 µL of the protein sample was subsequently added to the plate which was allowed to dry. 1 µL of matrix was finally added after evaporation of the protein sample. |

**Table S2** MALDI-TOF-MS sample preparation optimization results from 10 matrices combined with 5 different proteins, using the mix method for preparation.

| Matrix\Types of protein | Insulin | Cytochrome | Apomyoglobin | Aldolase | Albumin |
| --- | --- | --- | --- | --- | --- |
| SA | √ | √ | √ | √ | √ |
| CA | √ | √ | √ | √ |  |
| DHB | √ | √ | √ |  |  |
| FA | √ | √ | √ |  |  |
| HABA | √ |  | √ |  |  |
| CHAH | √ | √ |  |  |  |
| 9-AA | √ |  |  |  |  |
| THAP | √ |  |  |  |  |
| DHAP | √ | √ | √ |  |  |
| 1,8,9Anthractral | √ |  |  |  |  |

The “tick” sign indicates the detection of a particular protein.

**Table S3** MALDI-TOF-MS sample preparation optimization results from 10 matrices combined with 5 different proteins, using the overlay method for preparation.

| Matrix\Types of protein | Insulin | Cytochrome | Apomyoglobin | Aldolase | Albumin |
| --- | --- | --- | --- | --- | --- |
| SA | √ | √ | √ | √ |  |
| CA | √ | √ | √ |  |  |
| DHB | √ | √ | √ |  |  |
| FA | √ | √ | √ |  |  |
| HABA | √ |  |  |  |  |
| CHAH | √ |  |  |  |  |
| 9-AA | √ |  |  |  |  |
| THAP | √ | √ | √ |  |  |
| DHAP | √ |  |  |  |  |
| 1,8,9Anthractral |  |  |  |  |  |

The “tick” sign indicates the detection of a particular protein.

**Table S4** MALDI-TOF-MS sample preparation optimization results from 10 matrices combined with 5 different proteins, using the underlay method for preparation.

| Matrix\Types of protein | Insulin | Cytochrome | Apomyoglobin | Aldolase | Albumin |
| --- | --- | --- | --- | --- | --- |
| SA | √ |  |  |  |  |
| CA | √ |  |  |  |  |
| DHB |  |  |  |  |  |
| FA | √ | √ |  |  |  |
| HABA | √ |  |  |  |  |
| CHAH | √ |  |  |  |  |
| 9-AA | √ |  |  |  |  |
| THAP | √ |  |  |  |  |
| DHAP | √ |  |  |  |  |
| 1,8,9Anthractral |  |  |  |  |  |

The “tick” sign indicates the detection of a particular protein.

**Table S5** MALDI-TOF-MS sample preparation optimization results from 10 matrices combined with 5 different proteins, using the sandwich method for preparation.

| Matrix\Types of protein | Insulin | Cytochrome | Apomyoglobin | Aldolase | Albumin |
| --- | --- | --- | --- | --- | --- |
| SA | √ | √ | √ | √ |  |
| CA | √ | √ | √ |  |  |
| DHB | √ | √ | √ |  |  |
| FA | √ | √ | √ |  |  |
| HABA |  |  |  |  |  |
| CHAH | √ |  |  |  |  |
| 9-AA | √ |  |  |  |  |
| THAP | √ | √ | √ |  | √ |
| DHAP |  |  |  |  |  |
| 1,8,9Anthractral |  |  |  |  |  |

The “tick” sign indicates the detection of a particular protein.

**Table S6** Prediction accuracies of the 34 *Bacillus* strains using DAG-SVM with Linear kernel models.

The different colours represent the species level identifications.

**Table S7** Prediction accuracies of the 34 *Bacillus* strains using DAG-SVM with Jaccard kernel model.

The different colours represent the species level identifications.
